# Using a general practice research database to assess the spatio-temporal COVID-19 risk

**DOI:** 10.1186/s12875-024-02423-3

**Published:** 2024-05-21

**Authors:** Oana Petrof, Thomas Neyens, Bert Vaes, Arne Janssens, Christel Faes

**Affiliations:** 1 I-Biostat, Hasselt University, Diepenbeek, Belgium; 2 Leuven Biostatistics and Statistical Bioinformatics Centre (L-BioStat), KU Leuven, Leuven, Belgium; 3 Department of Public Health and Primary Care, KU Leuven, Leuven, Belgium

**Keywords:** Spatio-temporal methods, COVID-19, Comorbidities

## Abstract

**Background:**

In Flanders, general practitioners (GPs) were among the first ones to collect data regarding COVID-19 cases. Intego is a GPs’ morbidity registry in primary care with data collected from the electronic medical records from a sample of general practices. The Intego database contain elaborate information regarding patient characteristics, such as comorbidities. At the national level, the Belgian Public Health Institute (Sciensano) recorded all test-confirmed COVID-19 cases, but without other patient characteristics.

**Methods:**

Spatio and spatio-temporal analyses were used to analyse the spread of COVID-19 incidence at two levels of spatial aggregation: the municipality and the health sector levels. Our study goal was to compare spatio-temporal modelling results based on the Intego and Sciensano data, in order to see whether the Intego database is capable of detecting epidemiological trends similar to those in the Sciensano data. Comparable results would allow researchers to use these Intego data, and their wealth of patient information, to model COVID-19-related processes.

**Results:**

The two data sources provided comparable results. Being a male decreased the odds of having COVID-19 disease. The odds for the age categories (17,35], (35,65] and (65,110] of being a confirmed COVID-19 case were significantly higher than the odds for the age category [0,17]. In the Intego data, having one of the following comorbidities, i.e., chronic kidney disease, heart and vascular disease, and diabetes, was significantly associated with being a COVID-19 case, increasing the odds of being diagnosed with COVID-19.

**Conclusion:**

We were able to show how an alternative data source, the Intego data, can be used in a pandemic situation. We consider our findings useful for public health officials who plan intervention strategies aimed at monitor and control disease outbreaks such as that of COVID-19.

## Introduction

COVID-19 is a respiratory disease caused by a highly infectious corona virus, SARS-CoV-2 [1, 2], which has quickly spread across continents. Since the initial outbreak of the global pandemic, governments and governmental agencies around the world were responsible for the epidemiological follow-up of the COVID-19 epidemic. March 2020 is the date commonly referred to as the start of the epidemic in Belgium [3]. Given the rapid increase in COVID-19 cases, the Belgian government decided to implement a lockdown from March until April 2020, i. e., during the first wave of the epidemic (1 March 2020 until 21 June 2020) [4, 5]. Later, from October until April 2021, a second lockdown was imposed during the second wave (31 August 2020 until 14 February 2021) [4, 5].

It is crucial to have a reliable source of data to follow up the epidemic, where complex analyses such as spatio and spatio-temporal models can detect local outbreaks or local hotspots of the epidemic. In Belgium, the leading scientific institution in the epidemiology of infectious diseases, Sciensano is responsible for monitoring the epidemic evolution and assessing its consequences on the Belgian population health [6]. They did set up a new database during the pandemic, to collect information about the daily COVID-19 data new cases, hospitalizations, ICU admitted patients and deaths [7], together with their age, gender, and residential postal codes. Although this has been a very important database, it lacks further individual level information. Multiple comorbidities are associated with the COVID-19 disease progression [8–10], while the elderly population is more susceptible to the COVID-19 disease [8, 11]. Comorbidities often increase the probability of infection and represent a risk factor for COVID-19 patients [8, 10, 12]. Underlying diseases, such as hypertension, cardio-vascular diseases, diabetes, and asthma, have been reported as risk factors for COVID-19, increasing the mortality rate [9, 10, 12, 13]. Information on the comorbidities of the patients could not be collected in the federal database, but this information can easily be collected by general practitioners (GPs), since they follow up their patients in time. The Intego network represents a GP morbidity registry, implemented in Flanders, in which GPs continuously register information about their patients’ sociodemographic variables, diagnoses, clinical parameters, laboratory results and medication prescriptions [14]. Other European countries like the United Kingdom [15, 16] or the Netherlands [17] implemented a similar GP registry, given it’s added value.

The objective of this paper was to validate the Intego database by comparing the spatio-temporal analysis results with those based on the the federal COVID-19 data. The advantage of using the Intego study is the availability of individual level information data, which includes details of the patients, like comorbidities. In our study, results from the national COVID-19 database are considered as the “gold standard” showing the real spatio-temporal distribution of all COVID-19 cases in Flanders. We investigated if a GP morbidity registry could provide similar and, thus, reliable results in a pandemic situation,further adding important information on the association between COVID-19 and comorbidity.

## Methods

### Data sources

In the current study two data sources on COVID-19 were analyzed: (i) confirmed positive COVID-19 cases based on the Intego database, a registration network for participating family practices in Flanders [14] and (ii) reported test-confirmed cases in Belgium based on data provided by the Belgian Public Health Institute (Sciensano). The study period was chosen to cover the first two COVID-19 waves, from March 1st until November 30th 2020, before the start of the nationwide vaccination campaign in Belgium.

The Intego morbidity registration network collects data from GPs using the medical software program CareConnect® (Corilus, Namur, Belgium). On 31 December 2019, there were 16,722 active GP’s and 2,209 GP’s in training in Belgium, with 2,65 GPs per practice in Belgium [18]. During the study period, the Intego program included data from 104 public GP practices spread throughout Flanders, covering patients with varying socio-demographic and socio-economic backgrounds. The Intego GPs registered new COVID-19 diagnoses. We estimate the incidence of registered patients with a confirmed positive COVID-19 diagnosis as the ratio between the number of patients that tested positive and the patient population. We assume that the patient population is actually the yearly contact group [19], which consists of those patients who contacted their general practitioner at least once from December 1st 2019 until November 30th 2020. The Intego database includes for each patient the residential information (postal code), age, gender and presence/absence of the following comorbidities: asthma, chronic liver disease, chronic lung disease, chronic neurological disorder, chronic kidney disease, heart and vascular disease, diabetes, hypertension, immunodeficiency and obesity. All International Classification of Primary Care (ICPC) codes can be found in the Appendix, Table 4.

As a second data source, we used data from the Belgian population health institute (Sciensano, https://epistat.wiv-isp.be/covid/), responsible for the epidemiological follow-up of the COVID-19 epidemic in Belgium. They collect daily numbers on confirmed COVID-19 cases, hospitalizations and mortality. We used summary data on the number of cases per age group, gender and postal code residential information, who were diagnosed with COVID-19 between March 1st and November 30th 2020.

We defined the COVID-19 incidence as the proportion of COVID-19 positive cases divided by the population size. In this case, the Flanders’ population in 2019 is used to describe the “population at risk” per area.

The analysis was done at two different levels of areal aggregation: at the municipality level and at the health sector level. The 300 municipalities in Flanders are included in our analysis.

Health sectors, denoted as primary care zones (PCZs), create the basis for effective integrated care and services in a locality, each serving a community of approximately 75 000 - 125 000 inhabitants [20]. The role of these organizations include aligning the organization and provision of high-quality care and support; supporting local social policy; supporting profession-specific associations; supporting primary care professionals and the organization of multi- and interdisciplinary collaboration; and cooperating on the Flemish health objectives relating to prevention and propose their own objectives [20]. There are 59 health sectors in Flanders which were included in our study.

**Fig. 1:**
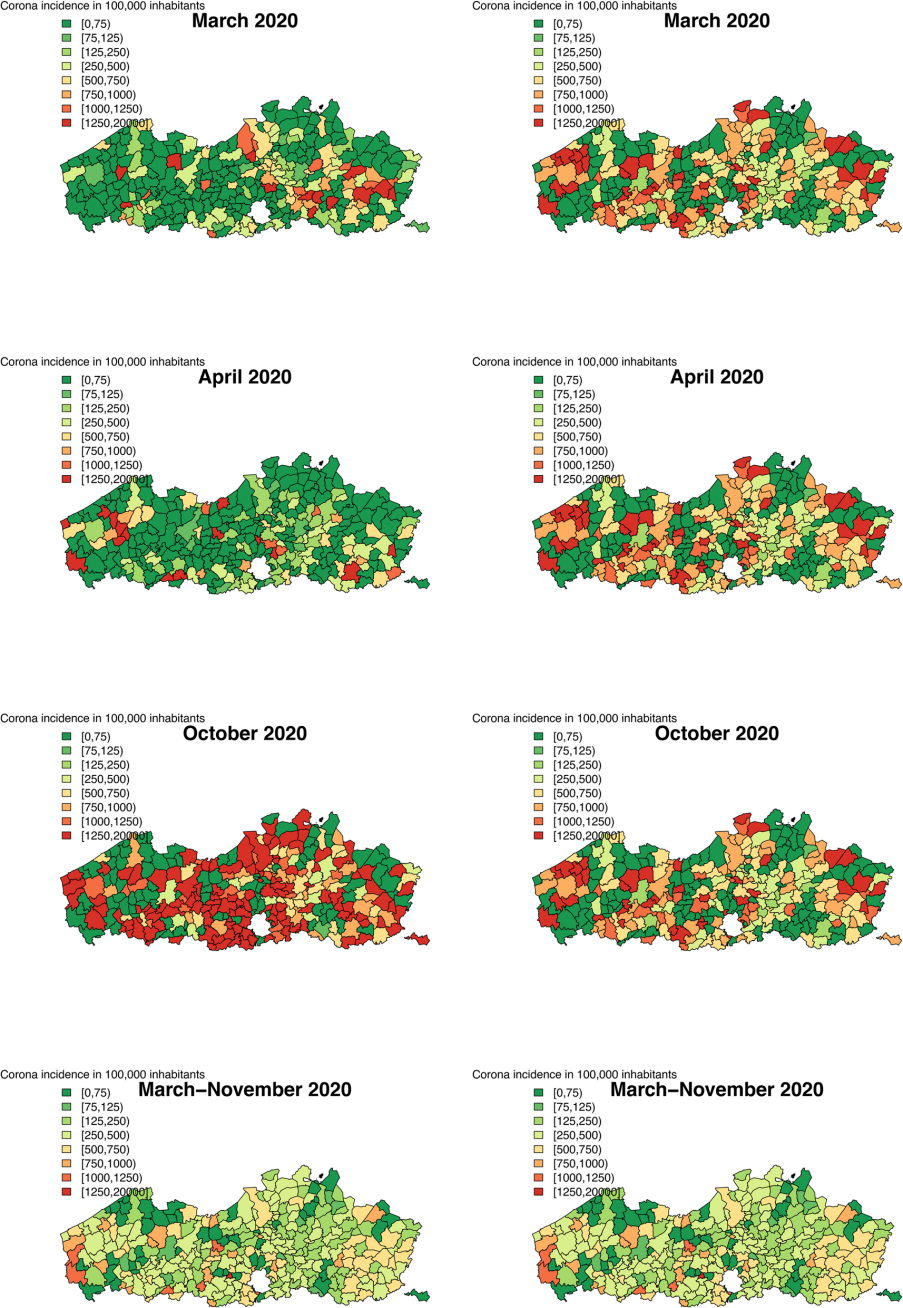
Incidence of positive COVID-19 cases in a population of 100,000 inhabitants using the Intego data (left) and the Sciensano data (right)

Looking at the observed incidences on Fig. 1 as well as in the Appendix on Figs. 11 and 12, we can see that overall trends are similar, but the maps based on the national database are much smoother as compared to the observed incidences based on the Intego database. This could be expected as the Intego database contains only information for a fraction of the Flemish population.

### Models

Disease mapping methods play an important role in understanding the spatial pattern of diseases and discovering areas characterized by unusually high or low risk [21–24]. These methods are commonly used for areal data, usually based on administrative boundaries, which are discrete in nature, as counts of diseases or deaths in each area [23]. We developed two models: (1) a validation model which uses only the age, gender, and residential information similar to the information available from the federal database, and (2) a comorbidity model using additional patient level information from the Intego database.

#### Validation model

Let’s assume the total number of confirmed COVID-19 cases $Y_{itga}$ in area *i* during month *t* with gender *g* in age group *a* have a binomial distribution

1 $$\begin{aligned} Y_{itga} \sim Binomial(\pi _{itga}, n_{iga}), \end{aligned}$$

where the parameter $n_{iga}$ denotes the number of people at risk in area *i* with gender *g* in age group *a*, which is constant during the entire time period analysed. Spatial and spatio-temporal models with various combinations of covariates and their interactions as well as different random effects for the spatial and temporal effects are used as depicted in Table 2, in Appendix. The interactions between different spatial and temporal random effects were mainly developed by Knorr-Held [25], but a good overview and applications can be found in the book written by Blangiardo & Cameletti [23]. They include spatially structured and unstructured random effects, a temporally structured effect (see Table 2 in Appendix). We used model selection to find the best fitting model, via the WAIC (Watanabe-Akaike Information Criterion, [26]) and the DIC (Deviance Information Criterion, [27]). However, the best fitting models, model 15 and 16, included the interaction of the covariates and the categorical time effects. The interpretation of these parameters did not make sense from a medical and statistical point of view. Scientific rationale and expert opinion were used to develop the second best fitting model for our data (model 18).

We introduced the spatio-temporal model found to be the best fitting model for our study. The probability $\pi _{itga}$ is modelled via a logit link, as follows:

2 $$\begin{aligned} logit(\pi _{itga}){} & {} = log(\pi _{itga}/(1 - \pi _{itga})) =\nonumber \\{} & {} = \beta _0 + \beta _1*gender_i + \beta _2*agegroup_{2i} + \beta _3*agegroup_{3i} + \beta _4*agegroup_{4i}+ \nonumber \\{} & {} + u_i + v_i + \gamma _t + \delta _{it}, \end{aligned}$$

where *gender* denotes a binary variable taking the value 1 when a patient is a male and 0 when a patient is a female; $agegroup_2$, $agegroup_3$ , and $agegroup_4$ are dummy variables which indicate whether patients belong to the age groups 17-35, 35-65, and +65 , respectively. The random effects term ${\textbf {u}}=(u_1, u_2, ..., u_n)$ accounts for the spatially correlated heterogeneity (CH) and the random effects term ${\textbf {v}}=(v_1, v_2, ..., v_n)$ for the uncorrelated heterogeneity (UH) at the areal level. The unstructured random effect is defined as $v_i \sim N(0,\sigma _v^2)$. The CH random effect is based on the full conditional distribution of the random effects $u_i$, an intrinsic conditional autoregressive (CAR) random effects term, as introduced by [28], as follows:

3 $$\begin{aligned}{}\left[u_i \pmb {|} u_j, j \ne i, \sigma _u^2\right] \sim N\left( \overline{u}_{i} , \sigma _i^2\right), \end{aligned}$$

where

$$\begin{aligned} \overline{u}_{i} = \frac{1}{\sum _{j} w_{i,j}} \sum \limits _{j} u_jw_{i,j} \end{aligned}$$

$$\begin{aligned} \sigma _i^2 = \frac{\sigma _u^2}{\sum _{j} w_{i,j}} \end{aligned}$$

and $\sigma _u^2$ is a variance parameter that controls the degree of smoothing, with the adjacency-based weights $w_{i,j}$. A binary indicator, is used based on sharing boundaries, with $w_{i,j}=1$ if the areas *i* and *j* were adjacent and 0 otherwise. The parameter $\gamma _t$ represents the temporally structured random effect, modeled dynamically using a random walk of order 1, defined as:

4 $$\begin{aligned} \gamma _t | \gamma _{t-1} \sim Normal\left(2\gamma _{t-1}, \sigma ^2\right). \end{aligned}$$

The parameter $\delta _{it}$ denotes the spatio-temporal interaction between an uncorrelated heterogeneity (UH) random effect and an unstructured time random effect. It represents unobserved covariates for each pixel (*i*, *t*) that do not have any structure in space and time [25], capturing variation which cannot be explained by the main effects of a model.

#### Comorbidities’ model

The analysis for the Intego data is done at both municipality and health sector level including comorbidities, using the model described by Eq. (2) adapted as follows:

5 $$\begin{aligned} logit(\pi _{itgac}){} & {} = \beta _0 + \beta _1*male_i + \beta _2*agegroup_{2i} + \beta _3*agegroup_{3i} + \beta _4*agegroup_{4i} + \nonumber \\{} & {} + \beta _5*comorbidity_i + u_i + v_i + \gamma _t + \delta _{it}, \end{aligned}$$

where *comorbidity* denotes a binary variable taking the value 1 when a comorbidity is present and 0 when a comorbidity is not present. The interpretation of the other parameters remains unchanged. Each individual comorbidity is included in the analysis in either:

- a univariate analysis, in which the significance of every comorbidity effect is tested separately, and
- a multivariate analysis, in which all significant comorbidities are included together in the final model.

### Parameter estimation

Spatial and spatio-temporal models are traditionally analyzed using Bayesian methods using Markov chain Monte Carlo (MCMC) simulation methods [23, 29, 30]. While the Bayesian approach can easily account for uncertainty in the estimates/predictions, can flexibly cope with complex model specifications and can easily deal with missing data, its main limitation is the computational burden [23]. Especially for large data collections, characterized by high spatial and temporal resolutions, the computing time needed to perform Bayesian inference via MCMC could take from hours or even days to compute accurate posterior marginals. To overcome this issue, we use the integrated nested Laplace approximations (INLA) [31]. This alternative estimation method gained popularity as an approximation procedure via numerical integration for Bayesian computing.

The analysis is performed using the R-INLA package [31] in R [32].

## Results

### Validation of the Intego database

The municipality analysis’ results are presented in this section, while the health sector analysis’ results are shown in the Appendix section. Both analyses lead to similar general conclusions. Significant effects were found for gender and age categories, with similar trends found as based on the national collected database data (Table 1). Based on the Intego data, the odds of being a confirmed COVID-19 case for males are 0.94 (0.90; 0.98) times the odds for females, when controlling for all other variables. In terms of percent change, we can say that the odds of being a confirmed COVID-19 case for males are 5.65% (2%; 10%) lower than the odds for females. The odds of being a confirmed COVID-19 case for the age categories (17,35], (35,65] and (65,110] are, respectively, 106% (91%; 121%), 83% (70%; 95%) and 10% (1.8%; 19.8%) higher than the odds for the age category [0,17], controlling for all other variables in the model.

Based on the Sciensano data, the odds of being a confirmed COVID-19 case for males are 17.54% (17.24%; 18.58%) lower than the odds for females. The odds for the age categories (17,35], (35,65] and (65,110] of being a confirmed COVID-19 case are 188% (187%; 196%), 135% (133%; 140%) and 141% (135%; 143%) higher than the odds for the age category [0,17], controlling for all other variables, which differs substantially from the results based on the Intego data.

**Table 1:** Parameter estimates at the municipality level for the Intego and Sciensano data

	Intego			Sciensano		
	Mean	2.50%	97.50%	Mean	2.50%	97.50%
Validation model						
$\hat{\beta _0}$	0.0010	0.0009	0.0011	0.0007	0.0007	0.0007
$\hat{\beta _1}$	0.9453	0.9076	0.9833	0.8209	0.8142	0.8276
$\hat{\beta _2}$	2.0650	1.9175	2.2150	2.9192	2.8740	2.9645
$\hat{\beta _3}$	1.8293	1.7042	1.9567	2.3683	2.3331	2.4035
$\hat{\beta _4}$	1.1077	1.0183	1.1986	2.3939	2.3551	2.4328
$\hat{v_i}$	0.1078	0.0422	0.1912	0.0007	0.0002	0.0014
$\hat{u_i}$	0.1270	0.0253	0.2581	0.0587	0.0447	0.0732
$\hat{\gamma _t}$	0.6738	0.2166	1.3050	2.5216	0.8301	4.6852
$\hat{\delta _{it}}$	0.3552	0.2907	0.4243	0.2562	0.2374	0.2744
Comorbidity model						
$\hat{\beta _0}$	0.0010	0.0009	0.0011			
$\hat{\beta _1}$	0.9371	0.8996	0.9749			
$\hat{\beta _2}$	2.0584	1.9113	2.2080			
$\hat{\beta _3}$	1.7821	1.6592	1.9071			
$\hat{\beta _4}$	0.9855	0.8999	1.0727			
$\hat{\beta _5}$ Chronic kidney disease	1.3026	1.1156	1.4937			
$\hat{\beta _6}$ Heart and vascular disease	1.1760	1.0929	1.2600			
$\hat{\beta _7}$ Diabetes	1.2087	1.1065	1.3122			
$\hat{v_i}$	0.0986	0.0323	0.1801			
$\hat{u_i}$	0.1470	0.0348	0.2975			
$\hat{\gamma _t}$	0.7086	0.1761	1.4036			
$\hat{\delta _{it}}$	0.3574	0.2846	0.4274			

### Comorbidities’ results

Three comorbidities had important, i.e., “significant”, effects based on the univariate analysis, thus were included in the multivariate analysis. The odds for the patients diagnosed with chronic kidney disease, heart and vascular disease and diabetes of being a confirmed COVID-19 case are 30% (11%; 49%), 17% (9%; 26%) and 20% (10%; 31%) higher than the odds of a patient without other comorbidities, controlling for all other variables.

The two random effects, the spatially correlated heterogeneity (CH) and the uncorrelated heterogeneity (UH), are not uniquely identifiable and only their sum is well identified by the data [33]. We interpret for every model the sum of these spatial random effects, while avoiding interpreting them separately. We observe a larger effect of the random effects components in the Intego analysis, as compared to the Sciensano analysis. For the Intego analysis, the variations of the random effects component were close for both analyses, with or without the comorbidities’ effects. The addition of the comorbidities as covariates in our model did not explain part of the variation.

The temporal structured effects over time, together with the 95% credible intervals, are plotted for Intego and Sciensano data, based on the two aggregation levels, municipality and health sector level (Fig. 5, in Appendix). Two waves of the pandemic can easily be observed in the months of March - April, 2020 and September - November, 2020. A slight disagreement was observed for the first wave between the two data sources. While the results based on the Sciensano database do not capture the cluster of increased risk present in the eastern part of Flanders, the Intego database results reflect the starting point of the epidemic in Belgium. Starting from May until September, a constant time trend effect can be observed for the probability of being diagnosed with COVID-19. This probability tremendously increased during the second Belgian wave, which lasted from September until November 2020, with both data sources being in agreement. It is clear that the results do not reflect the same incidence for this time period. More heterogeneity is present on the Intego compared to Sciensano results. Likely, this is the case, because the Intego database represents a subsample of the total COVID-19 cases, which the Sciensano database should contain.

**Fig. 2:**
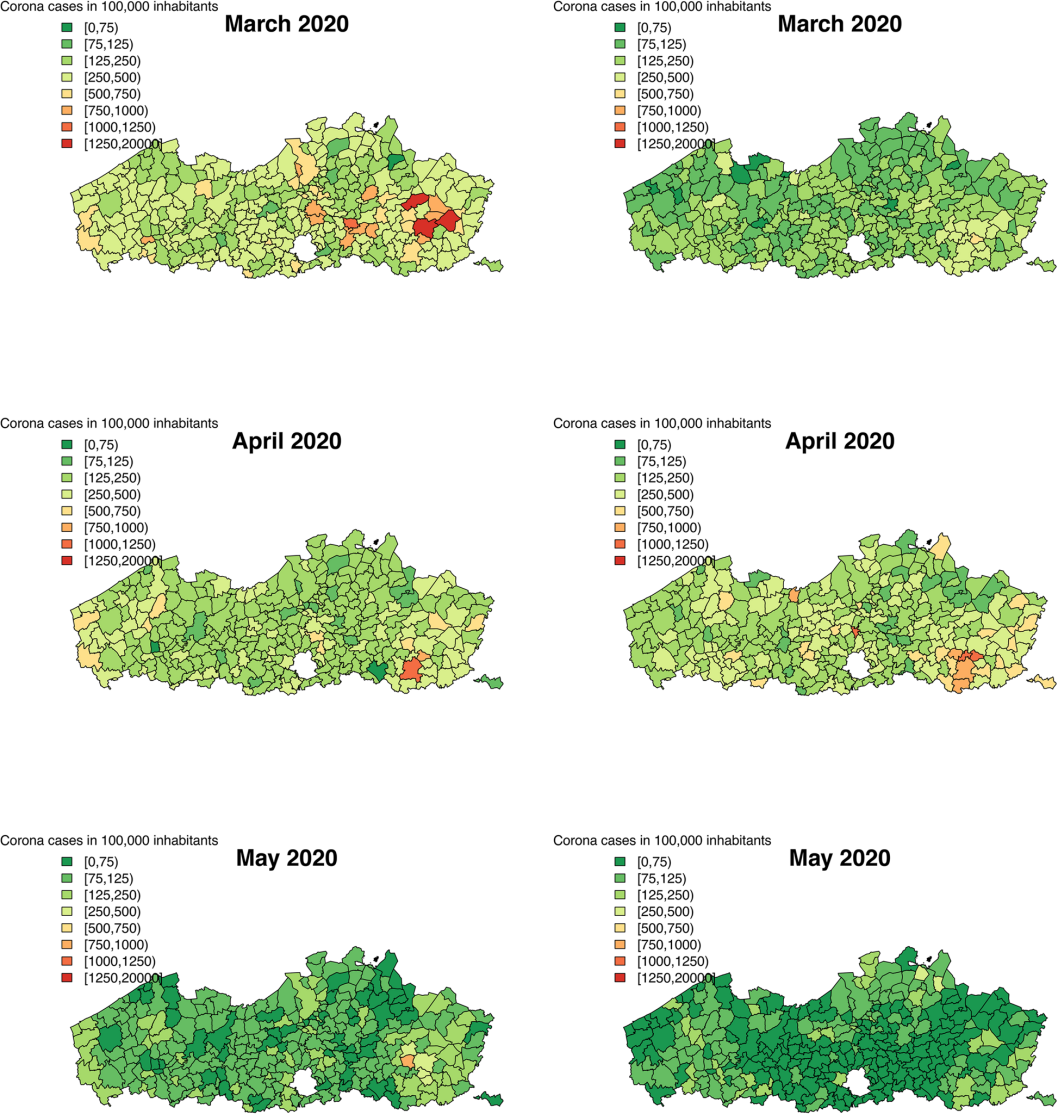
Predicted number of corona cases in a population of 100,000 inhabitants using the Intego data (left) and the Sciensano data (right)

**Fig. 3:**
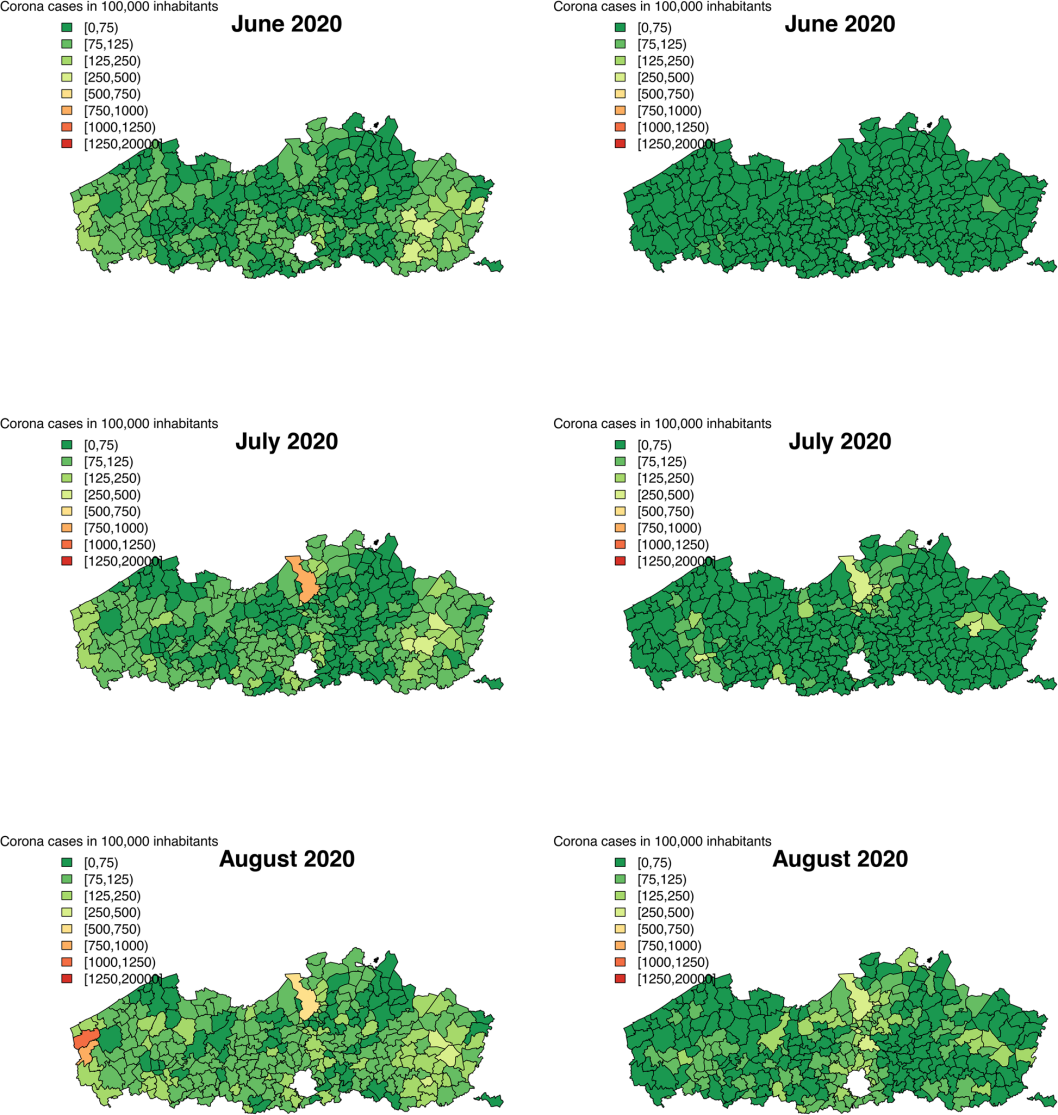
Predicted number of corona cases in a population of 100,000 inhabitants using the Intego data (left) and the Sciensano data (right)

**Fig. 4:**
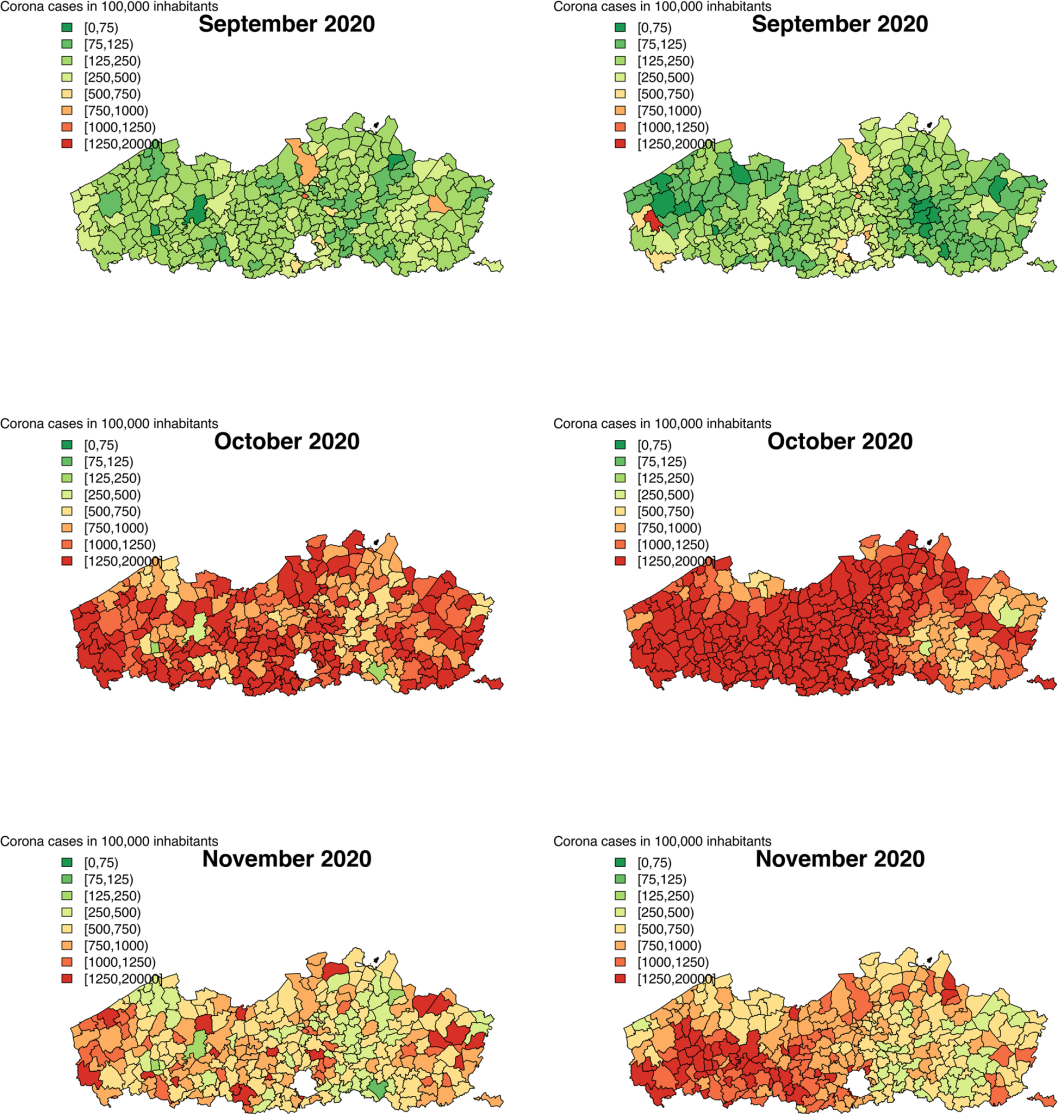
Predicted number of corona cases in a population of 100,000 inhabitants using the Intego data (left) and the Sciensano data (right)

**Fig. 5:**
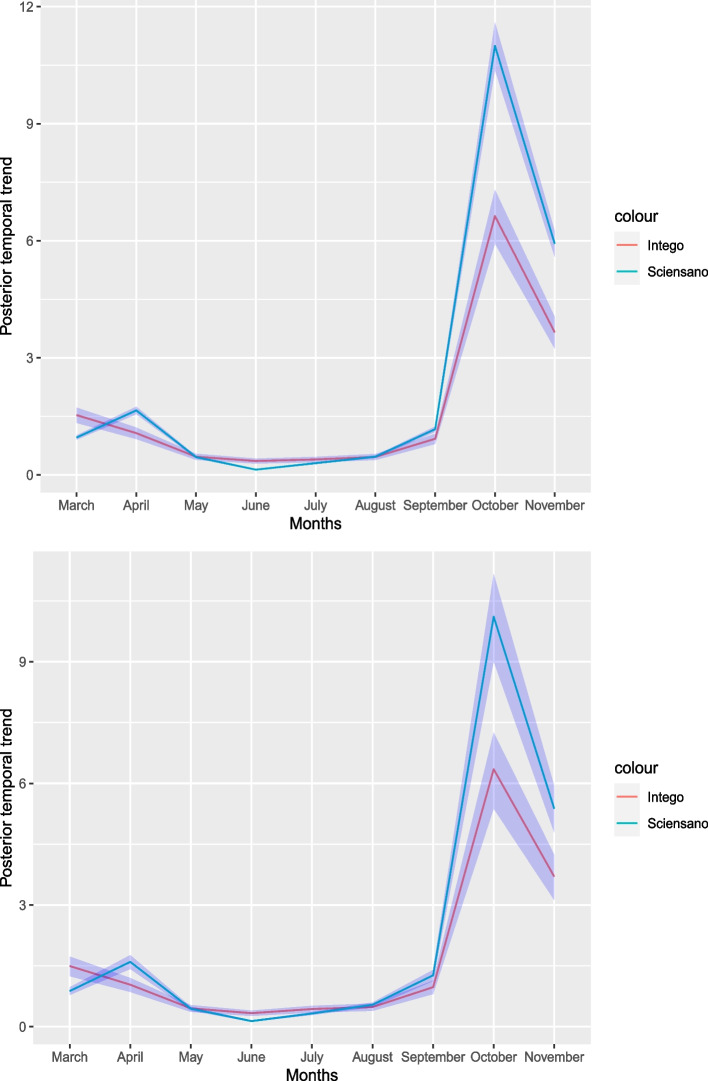
The temporal structured random effect with 95% credibility intervals based on the municipality level (top) and based on the health sector level (bottom)

**Fig. 6:**
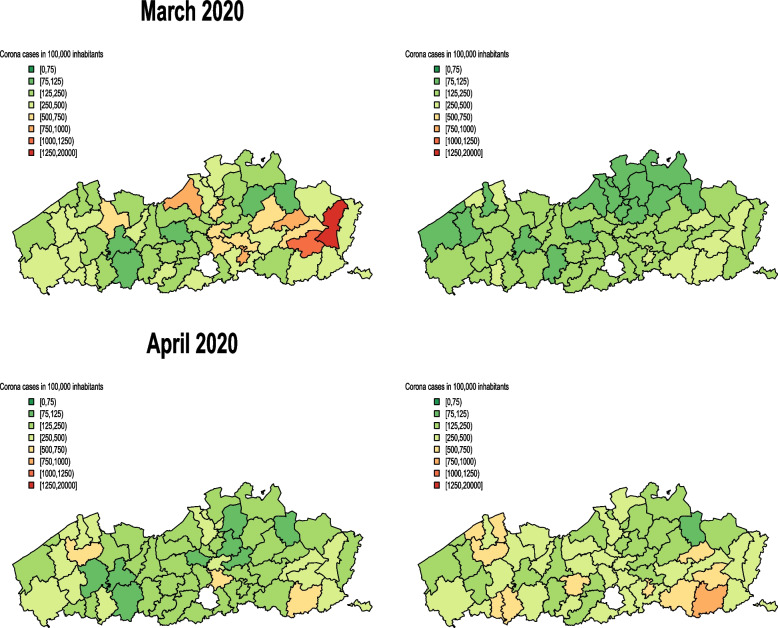
Predicted number of corona cases in a population of 100,000 inhabitants using the Intego data (left) and the Sciensano data (right) at the health sector level

**Fig. 7:**
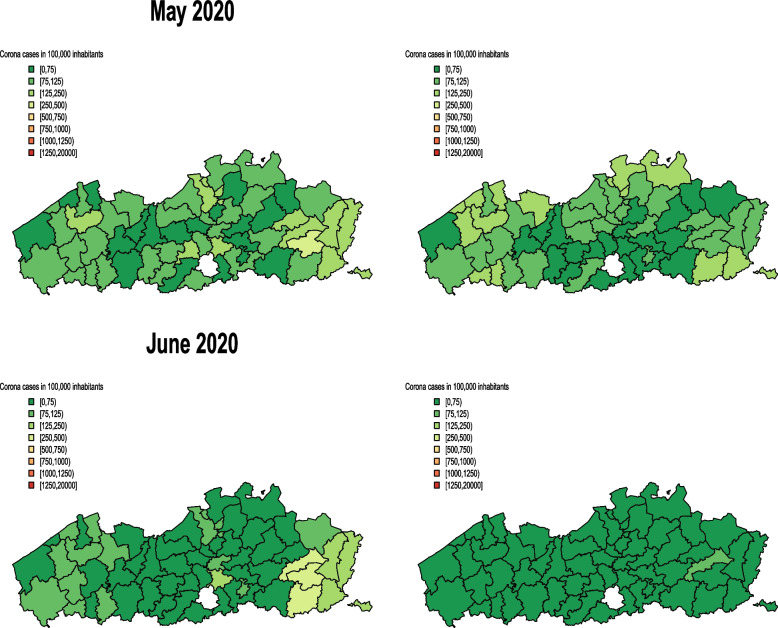
Predicted number of corona cases in a population of 100,000 inhabitants using the Intego data (left) and the Sciensano data (right) at the health sector level

**Fig. 8:**
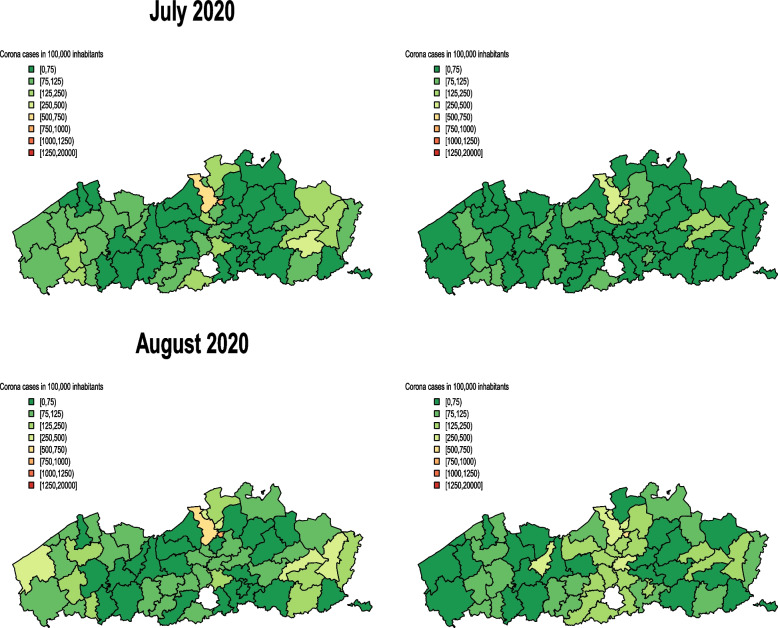
Predicted number of corona cases in a population of 100,000 inhabitants using the Intego data (left) and the Sciensano data (right) at the health sector level

**Fig. 9:**
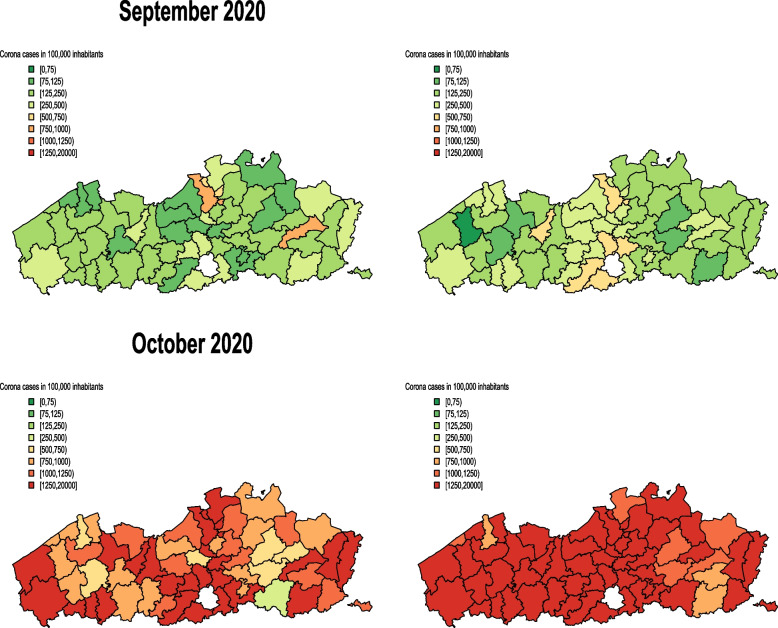
Predicted number of corona cases in a population of 100,000 inhabitants using the Intego data (left) and the Sciensano data (right) at the health sector level

**Fig. 10:**
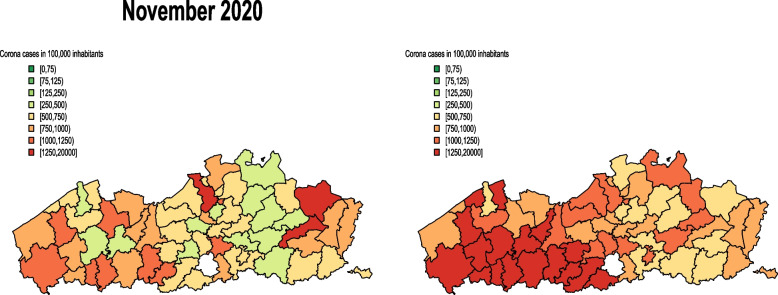
Predicted number of corona cases in a population of 100,000 inhabitants using the Intego data (left) and the Sciensano data (right) at the health sector level

**Fig. 11:**
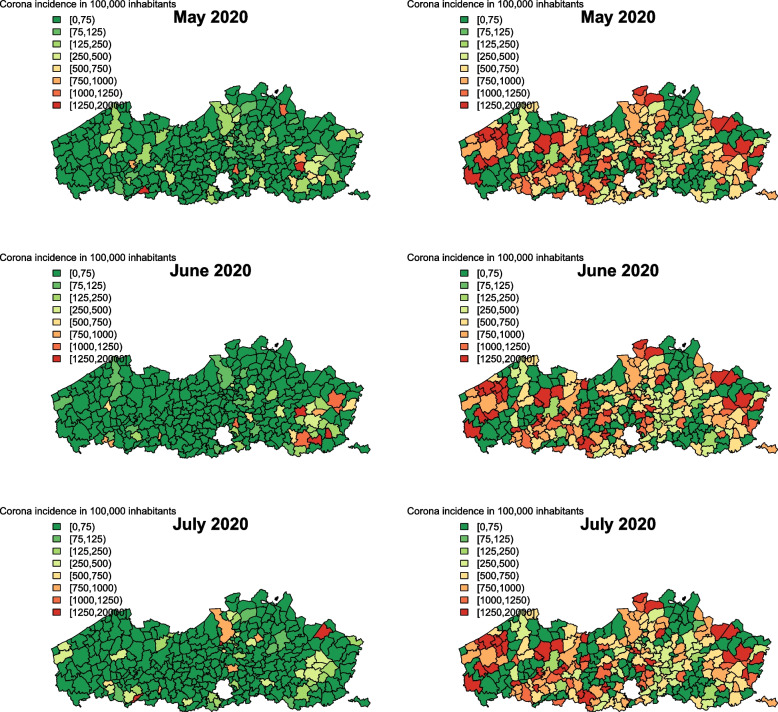
Incidence of positive COVID-19 cases in a population of 100,000 inhabitants using the Intego data (left) and the Sciensano data (right)

**Fig. 12:**
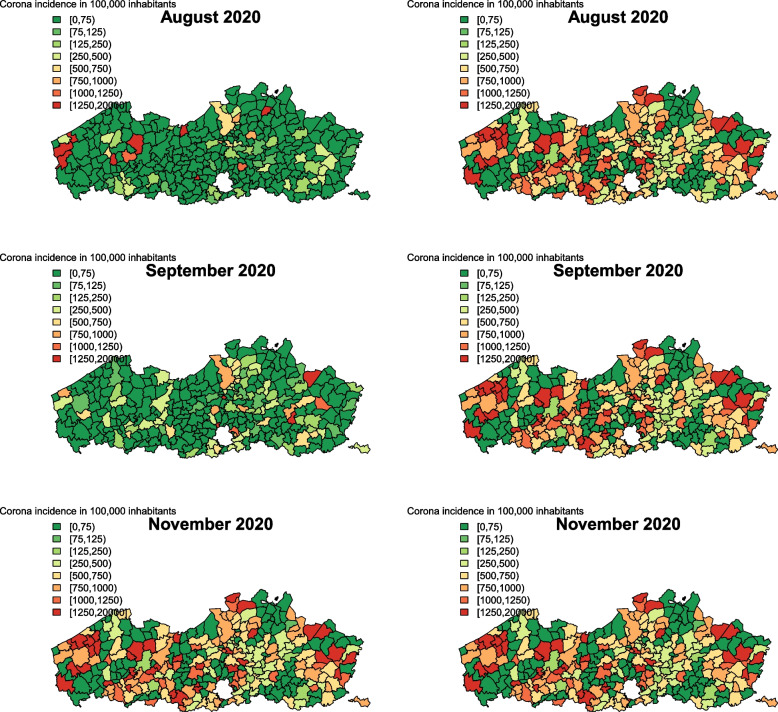
Incidence of positive COVID-19 cases in a population of 100,000 inhabitants using the Intego data (left) and the Sciensano data (right)

We calculated the predicted number of COVID-19 confirmed cases in a population of 100,000 inhabitants for both data sources and both levels of aggregation. Figures 2, 3 and 4 show the predicted number of cases per municipality per month, while Figs. 6, 7, 8, 9 and 10 in Appendix show the predicted number of cases per health sector per month. To make the predictions comparable between the two data sources, the Flanders’ population in 2019 is used to calculate the predicted incidence per area and month. Over the entire time period, it seems that the two data sources provide similar predicted number of cases per area and month, with slight differences due to variation. The two waves of the pandemic are present on these maps as well. For the first Belgian wave (March - April, 2020), a similar disagreement is present between the results of the Intego and Sciensano data analyses, as was seen for the temporal structured random effects. We note that this map of model-based predictions is smoother than those of the observed incidences (Figs. 1, 11 and 12 in Appendix), a direct effect of the modelling procedure that captures the mean spatial trend in incidences.

The maps of the predicted number of COVID-19 cases clearly show us the two pandemic waves, March-April 2020 and September-November 2020. During the first wave, a cluster of increased number of cases was located around Limburg province, in the Eastern part of Flanders. This part of the country was the first one to be affected by the COVID-19 disease. Over the entire time period, the results of the two analyses for the two databases are in agreement, with a small deviation during the first wave. This difference can be attributed to the difference in the coding system between the two databases in the beginning of the epidemic. From May until August, 2020, the number of cases was decreasing over the entire study region, most likely as a consequence for the strict lockdown measures imposed by the Belgian and Flemish government during the first pandemic wave. In September, the number of cases started to increase again, reaching a peak in October. Note that, likely due to a smaller sample size in the Intego database, we observe more variability of the maps compared to Sciensano results.

Age and gender have significant effects on the probability to have COVID-19. Males have a slightly lower probability to get COVID-19 compared to females. The effect for gender is larger based on the Sciensano database. A larger effect based on the Sciensano database can be seen for the age groups as well. The sample size of Intego data is much smaller (9,467 patients) compared to Sciensano data (235,066 individuals).

## Discussion

In this manuscript, a spatio-temporal model was used to investigate the distribution of COVID-19 cases in Flanders, Belgium based on two different databases. The Sciensano database was assumed to reflect the true spread of COVID-19 disease. Over the entire time period, the results of the two analyses for the two databases were in agreement, with a small deviation during the first wave. This difference can be attributed to the difference in the coding system between the two databases at the start of the Flemish epidemic. Using data from the Intego database, we examined if a GP morbidity registry could be used in a pandemic situation and we investigated if specific comorbidities had an influence on the COVID-19 disease progression. Our findings are in agreement with other studies, showing that patients with at least one of these comorbidities are more likely to be diagnosed with COVID-19 disease [8, 12, 34]. These results suggest that age, gender and comorbidities represent risk factors for critical patients with COVID-19 [8, 11].

The results of the health sector analyses presented in the Appendix section of our study are in agreement with the results at the municipality level. The health sector analyses’ results are important for policy makers since the COVID-19 policies are made at this level. Moreover, especially model-based results are useful for policy-makers, and often more than maps of observed incidences, as model-based estimates provide insight in mean trends across the region of interest, here Flanders.

Our study has a number of limitations. The amount of GPs included in the Intego project is relatively small compared to the total number of GPs in Flanders. Progress is being made since every year new GPs are joining this database, thus more data about more patients are collected. Every area has a different number of practices and every practice has a different number of GPs included in the Intego project. Moreover, the number of tests performed per area varies, but their availability might also differ temporally, due to changes in the production capacity of these tests. For instance, during the first pandemic wave, due to limited capacity, only a fraction of suspected Belgian COVID-19 patients has been tested to confirm SARS-CoV-2 infection [35]. Moreover, it should be noted that patients with a comorbidity may have been tested more often by their GP’s, as compared to a person without comorbidities. We did not correct for these factors in our analyses and future work should be done to correct for these factors.

The population at risk for the two analysis is different. In the Sciensano database, it consists of all Flanders residents, while in the Intego database, it only consists of individuals seeking treatment of any kind within a year. We assume here that the patients seeking treatment are a random subset of the total population of Flanders, but that assumption may not be valid, as socioeconomically deprived individuals are known to adopt tendencies to visit GPs that are different from those of the rest of the population.

The most important advantage of using the Intego database is the ability to use a GP morbidity registry for a cost-effective and timely investigation of the dynamics in an epidemic. Since the Intego database collects data on many pathogens, it can be used to investigate the disease risk of other epidemics, such as flu, gasto-enteritis, RSV infections . Thus, we can use a GP morbidity registry as an alternative to predict and monitor an epidemic, with the advantage of the availability of detailed patient information. Next to these advantages, the Intego database is continuously updated, data are added on a daily basis, and more GPs and practices start to use the database, effectively adding more patients into it.

As part of future work, a joint analysis of both databases will be investigated which may result in more precise estimates, as the simultaneous modelling of both diseases allows both processes to borrow information from each other. This can be implemented via the use of, e.g., correlated random-effects structures [36, 37] , which allow for shared stochasticity.

In conclusion, we were able to show how an alternative data source, the Intego data, can be used in a pandemic situation. We consider our findings useful for public health officials who plan intervention strategies aimed at bringing disease outbreaks such as the COVID-19 outbreak under control as well as to monitor disease outbreaks.

## Data Availability

The datasets generated and analysed during the current study are not publicly available due to inclusion of protected health information but can be made available subsequent to further de-identification upon reasonable request to the corresponding author (OP).

## Appendix

**Table 2:** Model selection results based on WAIC and DIC values. Covariates effects: gender (G), age groups (A), comorbitidy (C), categorical time effect per month (M) and their interactions. Random effects: spatially correlated heterogeneity ($u_i$), spatially uncorrelated heterogeneity ($v_i$), temporally structured effect modeled dynamically using a random walk of order 1 ($\gamma _{t1}$), temporally structured effect modeled dynamically using a random walk of order 2 ($\gamma _{t2}$), temporally structured effect modeled dynamically using an autoregressive random effect ($\gamma _{t3}$), unstructured temporal effect $\theta _i$, spatio-temporal interaction between a spatially uncorrelated heterogeneity random effect and an unstructured time random effect ($\delta _{it}$) and spatio-temporal interaction between a spatially uncorrelated heterogeneity random effect and a temporally structured effect modeled dynamically using a random walk of order 1 ($\phi _{it}$). Blangiardo & Cameletti [23] provide explanations and applications of various spatio-temporal interactions

Model	Covariate effects										Random effects								WAIC	DIC
	G	A	C	M	G*A	G*C	A*C	G*M	A*M	C*M	$u_i$	$v_i$	$\gamma _{t1}$	$\gamma _{t2}$	$\gamma _{t3}$	$\theta _i$	$\delta _{it}$	$\phi _{it}$		
1	$\checkmark$	$\checkmark$	$\checkmark$	$\checkmark$								$\checkmark$					$\checkmark$		22436.06	22360.80
2	$\checkmark$	$\checkmark$	$\checkmark$	$\checkmark$							$\checkmark$	$\checkmark$					$\checkmark$		22423.97	22347.64
3	$\checkmark$	$\checkmark$	$\checkmark$									$\checkmark$	$\checkmark$						22966.20	22901.82
4	$\checkmark$	$\checkmark$	$\checkmark$									$\checkmark$		$\checkmark$					22966.23	22901.80
5	$\checkmark$	$\checkmark$	$\checkmark$									$\checkmark$			$\checkmark$				22966.17	22901.82
6	$\checkmark$	$\checkmark$	$\checkmark$								$\checkmark$	$\checkmark$	$\checkmark$			$\checkmark$	$\checkmark$		21247.00	21180.36
7	$\checkmark$	$\checkmark$	$\checkmark$		$\checkmark$						$\checkmark$	$\checkmark$	$\checkmark$			$\checkmark$	$\checkmark$		21242.44	21176.52
8	$\checkmark$	$\checkmark$	$\checkmark$			$\checkmark$					$\checkmark$	$\checkmark$	$\checkmark$			$\checkmark$	$\checkmark$		21238.16	21170.81
9	$\checkmark$	$\checkmark$	$\checkmark$				$\checkmark$				$\checkmark$	$\checkmark$	$\checkmark$			$\checkmark$	$\checkmark$		21223.14	21155.51
10	$\checkmark$	$\checkmark$	$\checkmark$		$\checkmark$	$\checkmark$	$\checkmark$				$\checkmark$	$\checkmark$	$\checkmark$			$\checkmark$	$\checkmark$		21232.70	21163.13
11	$\checkmark$	$\checkmark$	$\checkmark$					$\checkmark$			$\checkmark$	$\checkmark$	$\checkmark$				$\checkmark$		21200.66	21132.34
12	$\checkmark$	$\checkmark$	$\checkmark$						$\checkmark$		$\checkmark$	$\checkmark$	$\checkmark$				$\checkmark$		20679.03	20637.31
13	$\checkmark$	$\checkmark$	$\checkmark$							$\checkmark$	$\checkmark$	$\checkmark$	$\checkmark$				$\checkmark$		21096.19	21036.21
14	$\checkmark$	$\checkmark$	$\checkmark$					$\checkmark$	$\checkmark$		$\checkmark$	$\checkmark$	$\checkmark$				$\checkmark$		20647.02	20605.71
15	$\checkmark$	$\checkmark$	$\checkmark$					$\checkmark$	$\checkmark$	$\checkmark$	$\checkmark$	$\checkmark$	$\checkmark$				$\checkmark$		20627.03	20589.03
16	$\checkmark$	$\checkmark$	$\checkmark$					$\checkmark$	$\checkmark$	$\checkmark$	$\checkmark$	$\checkmark$	$\checkmark$			$\checkmark$	$\checkmark$		20625.85	20586.26
17	$\checkmark$	$\checkmark$	$\checkmark$								$\checkmark$	$\checkmark$	$\checkmark$			$\checkmark$	$\checkmark$		21234.85	21168.21
18	$\checkmark$	$\checkmark$	$\checkmark$								$\checkmark$	$\checkmark$	$\checkmark$				$\checkmark$		21235.67	21169.81
19	$\checkmark$	$\checkmark$	$\checkmark$									$\checkmark$	$\checkmark$					$\checkmark$	27145.73	27344.24

**Table 3:** Parameter estimates (odds ratios) at the health sector level for the Intego and Sciensano data with and without comorbidities

	Intego				Sciensano			
	Mean	SD	2.50%	97.50%	Mean	SD	2.50%	97.50%
$\hat{\beta _0}$	0.0010	0.0001	0.0009	0.0011	0.0006	0.0000	0.0005	0.0006
$\hat{\beta _1}$	0.9453	0.0193	0.9076	0.9833	0.8198	0.0034	0.8131	0.8265
$\hat{\beta _2}$	2.0650	0.0759	1.9175	2.2150	2.9268	0.0231	2.8815	2.9722
$\hat{\beta _3}$	1.8293	0.0644	1.7042	1.9567	2.3598	0.0179	2.3248	2.3949
$\hat{\beta _4}$	1.1076	0.0460	1.0183	1.1986	2.3798	0.0197	2.3412	2.4184
$\hat{v_i}$	0.0932	0.0381	0.0315	0.1690	0.0545	0.0155	0.0315	0.16900
$\hat{u_i}$	0.1540	0.0735	0.0427	0.3002	0.0016	0.0032	0.0427	0.3002
$\hat{\gamma _t}$	0.6978	0.3579	0.2070	1.4112	1.2672	0.6164	0.2070	1.4112
$\hat{\delta _{it}}$	0.3559	0.0335	0.2918	0.4227	0.1985	0.0138	0.2918	0.4227
$\hat{\beta _0}$	0.0012	0.0001	0.0010	0.0013				
$\hat{\beta _1}$	0.9356	0.0191	0.8982	0.9733				
$\hat{\beta _2}$	2.0756	0.0763	1.9272	2.2265				
$\hat{\beta _3}$	1.7716	0.0628	1.6494	1.8958				
$\hat{\beta _4}$	0.9710	0.0433	0.8868	1.0567				
$\hat{\beta _5}$ (Chronic nierziekte)	1.3285	0.0981	1.1383	1.5228				
$\hat{\beta _6}$ (Harten vaatziekten)	1.1921	0.0431	1.1080	1.2772				
$\hat{\beta _7}$ (Diabetes)	1.2153	0.0527	1.1126	1.3193				
$\hat{v_i}$	0.1560	0.0426	0.0843	0.2425				
$\hat{u_i}$	0.0020	0.0045	0.0001	0.0069				
$\hat{\gamma _t}$	0.6789	0.3376	0.1879	1.3507				
$\hat{\delta _{it}}$	0.3028	0.0316	0.2425	0.3657				

**Table 4:** The International Classification of Primary Care (ICPC) codes per comorbidity

Comorbidity	ICPC codes
Asthma	R96
Chronic liver disease	D97
Chronic lung disease	R79, R84, R95
Chronic neurological disorder	N74, N86, N87, N88
Chronic kidney disease	U99
Diabetes	T89, T90
Heart and vascular disease	K74, K75, K76, K77, K78, K80, K82,
	K83, K84, K89, K90, K91, K92
Hypertension	K86, K87
Immunodeficiency	B72, B73, B74, B90
Obesity	T82
